# Supplementary material for: A Critical View of the Application of the APEX Software (Aqueous Photochemistry of Environmentally-Occurring Xenobiotics) to Predict Photoreaction Kinetics in Surface Freshwaters
Source: Molecules. 2019 Dec 18;25(1):9. doi: 10.3390/molecules25010009 (PMC7017383; doi:10.3390/molecules25010009)
Supplement: Supplementary file 1 [file molecules-25-00009-s001.zip › 3.2.4_gcc-4.4.0.zip!/3.2.4_gcc-4.4.0/3.2.4_gcc-4.4.0/doc/octave/html/interpreter/Command-Line-Editing.html]

Command Line Editing - Untitled


Next: Errors,
Previous: Getting Help,
Up: Getting Started


---

### 2.4 Command Line Editing

Octave uses the GNU Readline library to provide an extensive set of
command-line editing and history features. Only the most common
features are described in this manual. In addition, all of the editing
functions can be bound to different key strokes at the user's discretion.
This manual assumes no changes from the default Emacs bindings. See the GNU
Readline Library manual for more information on customizing Readline and
for a complete feature list.

To insert printing characters (letters, digits, symbols, etc.), simply
type the character. Octave will insert the character at the cursor and
advance the cursor forward.

Many of the command-line editing functions operate using control
characters. For example, the character `Control-a` moves the cursor
to the beginning of the line. To type `C-a`, hold down <CTRL>
and then press <a>. In the following sections, control characters
such as `Control-a` are written as `C-a`.

Another set of command-line editing functions use Meta characters. To
type `M-u`, hold down the <META> key and press <u>. Depending
on the keyboard, the <META> key may be labeled <ALT> or
even <WINDOWS>. If your terminal does not have a <META> key, you
can still type Meta characters using two-character sequences starting
with `ESC`. Thus, to enter `M-u`, you would type
<ESC> <u>. The `ESC` character sequences are also allowed on
terminals with real Meta keys. In the following sections, Meta
characters such as `Meta-u` are written as `M-u`.

- Cursor Motion- Killing and Yanking- Commands For Text- Commands For Completion- Commands For History- Customizing readline- Customizing the Prompt- Diary and Echo Commands
